# Effect of adrenaline on serum mid‐regional pro‐atrial natriuretic peptide and central blood volume

**DOI:** 10.1113/EP090516

**Published:** 2022-08-21

**Authors:** Casper Sejersen, Jonathan J. Bjerre‐Bastos, Jens P. Goetze, Henning B. Nielsen, Asger R. Bihlet, Niels H. Secher

**Affiliations:** ^1^ Department of Anaesthesia Rigshospitalet Institute for Clinical Medicine University of Copenhagen Copenhagen Denmark; ^2^ Department of Biomedical Sciences Faculty of Health and Medical Sciences University of Copenhagen Copenhagen Denmark; ^3^ NBCD A/S Herlev Denmark; ^4^ Department of Clinical Biochemistry Rigshospitalet Institute for Clinical Medicine University of Copenhagen Copenhagen Denmark; ^5^ Department of Biomedical Sciences Faculty of Health University of Copenhagen Copenhagen Denmark; ^6^ Department of Anaesthesia Zealand University Hospital Roskilde Institute for Clinical Medicine University of Copenhagen Copenhagen Denmark; ^7^ Department of Nutrition Exercise and Sports Faculty of Science University of Copenhagen Copenhagen Denmark

**Keywords:** adrenaline, central blood volume, mid‐regional pro‐atrial natriuretic peptide

## Abstract

**New Findings:**

- **What is the central question in this study?**
  Atrial natriuretic peptide (ANP) is secreted in response to atrial wall distension and thus allows for evaluation, albeit indirect, of the central blood volume. Adrenaline has chronotropic and inotropic effects. We evaluated whether the chronotropic and inotropic effects of adrenaline were reflected in mid‐regional proANP.
- **What is the main finding and its importance?**
  Central blood volume remained stable with infusion of adrenaline and yet mid‐regional proANP increased. Thus, the chronotropic and inotropic state of the heart or adrenaline directly induces release of ANP variants from the myocytes.

**Abstract:**

Atrial natriuretic peptide (ANP) has vasodilatory, natriuretic and diuretic properties. It is secreted in response to atrial wall distension and thereby provides an indirect evaluation of central blood volume (CBV). Adrenaline has chronotropic and inotropic effects that increase cardiac output. In the present study, we evaluated whether these effects were influenced by an increase in CBV and reflected in mid‐regional proANP (MR‐proANP) concentrations in the circulation, a stable proxy marker of bioactive ANP. Changes in CBV were evaluated by thoracic electrical admittance and haemodynamic variables monitored by pulse‐contour analysis during two intervals with graded infusion of adrenaline. Adrenaline infusion increased heart rate (by 33 ± 18%) and stroke volume (by 6 ± 13%), hence cardiac output (by 42 ± 23%; all *P* < 0.05). The increase in cardiac output did not result from an increase in CBV, because thoracic electrical admittance remained stable (−3 ± 17%; *P* = 0.230). Serum MR‐proANP concentrations were increased (by 26 ± 25%; *P* < 0.001) by adrenaline infusion and remained elevated 60 min postinfusion. We conclude that MR‐proANP in the circulation is affected not only by CBV, but also by increased chronotropy/inotropy of the heart, or that adrenaline directly induces release of ANP variants from the myocytes.

## 1 INTRODUCTION

Atrial natriuretic peptide (ANP) is a peptide with potent vasodilatory effects besides its diuretic and natriuretic properties. In healthy humans, ANP is secreted in response to atrial wall distension (Clerico et al., 2011), predominantly in the right atrium (Seul et al., 1992; Wong et al., 1988). Measurement of ANP in the circulation permits evaluation, albeit indirect, of the central blood volume (CBV), as illustrated, for example, by a marked decrease when preload to the heart is reduced by head‐up tilt (Matzen et al., 1990). Conversely, plasma ANP concentrations increase when atrial filling is enhanced during blood volume expansion (Legault et al., 1992), such as during exercise (Yoshiga et al., 2019). In support, there is an inverse relationship between plasma ANP and central venous pressure (CVP) during positive‐pressure breathing (Schütten et al., 1990), and ANP concentration in plasma, rather than CVP, is correlated with the reduction in CBV during hyperthermia (Vogelsang et al., 2012).

Despite atrial stretch being the major stimulus for ANP secretion, that is, by manipulating CBV, infusion of adrenaline increases plasma ANP in healthy volunteers (Morrow et al., 1989; Sanfield et al., 1987; Tunny et al., 1988). Adrenaline is known for its chronotropic and inotropic effects on the ventricles and the atria (Lemoine et al., 1988; Molenaar et al., 2007). Moderate intravenous infusion of adrenaline raises heart rate (HR) and systolic blood pressure, while diastolic blood pressure falls, making mean arterial pressure (MAP) change little with an increase in cardiac output (CO) and stroke volume (SV), hence total peripheral resistance decreases (Barcroft & Konzett, 1949). However, the increase in SV with adrenaline infusion does not seem to be the result of increased venous return to the heart, because there is no change in the diastolic heart volume (Kjellberg et al., 1952). Thus, it could be that ANP is released in response to increased cardiac contractility in addition to atrial distension as revealed by CBV. The increase in SV and CO during adrenaline infusion can be reflected by pulse‐contour analysis (Niemann et al., 2019; Rokamp et al., 2017), which uses deviations in arterial pressure determined either non‐invasively or invasively. Non‐invasively derived SV and CO are comparable to those derived by invasive monitoring during cardiothoracic surgery (Martina et al., 2012; Truijen et al., 2018), during orthostasis (Harms et al., 1999), in cardiovascular disease (Bogert et al., 2010) and in septic shock patients (Jellema et al., 1999).

We evaluated the response of serum mid‐regional proANP_(53–90)_ (MR‐proANP), a stable variant of ANP, to adrenaline infusion in middle‐aged to elderly humans and to changes in CBV by thoracic electrical admittance (TEA) with simultaneous recording of haemodynamic variables with pulse‐contour analysis.

## 2 METHODS

### 2.1 Ethical approval

The study was approved by the Danish national ethics committee (H‐20026057) and conducted in accordance with standards of the *Declaration of Helsinki* and performed in a subgroup of patients from a study on osteoarthritis, EFEX‐OA‐02, registered at clinicaltrials.org (registration no. NCT04542668). Written informed consent was obtained from all participants after verbal and written explanation of the study.

### 2.2 Participants

The main inclusion criteria were relevant to a clinical population and were as follows: age 40–75 years, bodyweight 50–100 kg and body mass index 18.5–35.0 kg/m^2^. Exclusion criteria were as follows: treatment with β‐blockers, monoamine oxidase inhibitors, systemic corticosteroids, vitamin K antagonists, new oral anticoagulants or heparin; systemic infection; inflammatory immune or autoimmune disease; any sign of previous or current cardiovascular disease; or being an athlete or highly trained individual.

### 2.3 Experimental protocol

**FIGURE 1:**
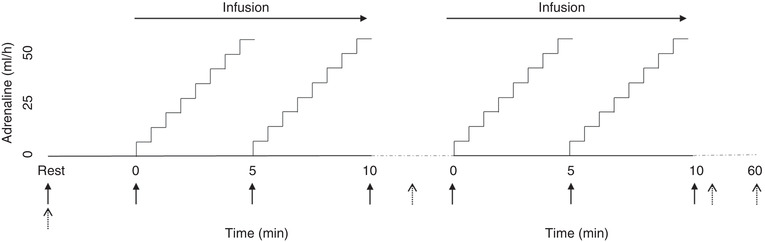
Experimental protocol. The two times two periods of adrenaline infusion were separated by ∼3 min. Solid arrows indicate the time for data analysis, and dotted arrows indicate time points for blood sampling

On intervention days, subjects refrained from caffeinated beverages and fasted for ≥6 h before visiting the laboratory. The subjects rested for 30 min. Then 0.06 mg/kg of adrenaline in 50 ml of saline solution was administered. With the participants semi‐recumbent (30° from the horizontal) and under ECG monitoring, adrenaline was administered through a forearm 20‐gauge intravenous catheter using an Infusomat Space (B. Braun, Melsungen, Germany). Adrenaline was administered in four 5‐min progressive intervals, with the infusion rate being increased gradually, while considering the subjective and objective wellbeing of the subjects. The infusion was increased gradually over each 5‐min interval in an attempt to mimic the cardiovascular stress during running and cycling to increase HR to ≥80% of the estimated heart rate reserve (Bjerre‐Bastos et al., 2022). After the first and third intervals, the infusion was paused for ∼1 min to reset resting cardiovascular variables, and the infusion was stopped after the second interval for blood collection. Blood samples were collected at rest, midway (i.e., after the second interval), after completion (i.e., after the fourth interval) and 1 h postinfusion for the determination of serum MR‐proANP and blood gas variables (Figure 1).

### 2.4 Measurements

#### 2.4.1 Haemodynamic variables

Arterial pressure and HR were recorded using photo‐plethysmography (Nexfin; BMEYE, Amsterdam, The Netherlands) with the finger cuff on the third middle phalanx, and a heart reference sensor was mounted to report values at the level of the heart. A pulse contour method (Nexfin CO‐trek; BMEYE) adapted for age, sex, height and weight (Bogert et al., 2010; Truijen et al., 2018) provided left ventricular SV. The CO was calculated as SV multiplied by HR, and systemic vascular resistance (SVR) was defined as the ratio of MAP to CO.

#### 2.4.2 Thoracic electrical admittance

The participants were instrumented with a pair of electrodes (N‐00‐25; Ambu, Ballerup, Denmark) placed on the right sternocleidomastoid muscle and another pair high in the left mid‐axillary line, with each pair separated by ∼5 cm, to estimate changes in CBV by TEA. Evaluation of TEA was based on an excitation current of 200 μA at 1.5 and 100 kHz (C‐Guard, Danmeter, Denmark), with the outer electrodes providing the current and the inner pair determining TEA. The low‐frequency current does not penetrate the cell lipid membrane readily and thus reflects the extracellular volume. Conversely, the high‐frequency current is correlated with total (regional) body water because it penetrates the cell membrane (Cai et al., 2000b). Changes in the difference between TEA (1/impedance, IDX) with a low‐ and a high‐frequency current are taken to reflect changes in intracellular volume, hence, presumably, in the regional red cell volume (Cai et al., 2000a, 2000b). In support of this, there is an almost perfect correlation between TEA and haemorrhage/reperfusion in pigs (Krantz et al., 2000), and TEA is sensitive to manipulation of CBV during, for example, simulated haemorrhage by head‐up tilt (Matzen et al., 1991; Van Lieshout et al., 2005) and lower‐body negative pressure (Cai et al., 2000b).

#### 2.4.3 Blood sampling

Venous blood samples were collected into 2 ml EDTA vacutainers for the determination of serum MR‐proANP. Measurement of MR‐proANP (amino acids 53–90) was chosen instead of ANP (amino acids 98–128) because ANP has a half‐life of only 2–3 min (Potter, 2011), whereas the MR‐proANP is suggested to be excreted in equimolar amounts to ANP but with a longer half‐life (Yagmur et al., 2019). After collection, the samples were coagulated for 30 min, centrifuged at 1610 *g* for 15 min at 4°C, and the serum samples were stored at −80°C until analysis.

Serum MR‐proANP was determined using an automated method from Thermo‐Fisher (the Kryptor Plus platform) and the sandwich immunoassay validated with excellent performance (Hunter et al., 2011). Also, venous blood was collected in a heparinized syringe (Pico; Radiometer, Brønshøj, Denmark) and immediately analysed for blood gas variables (ABL800 FLEX; Radiometer).

### 2.5 Statistics

Data are presented as the mean ± SD after the raw data were inspected and heart beats without apparent artefacts selected. A Shapiro–Wilk test was used to evaluate data distribution and a one‐way ANOVA with repeated measures to identify changes. Deviations from rest were identified using the Bonferroni post hoc test, with statistical significance set at *P* < 0.05, using SPSS statistics v.26 (IMB, Armonk, NY, USA). To evaluate changes over time, a 30 s average was used from rest and during the beginning and end of each infusion interval for cardiovascular variables and 15 s average for TEA. For overall evaluation, the last 1 min of rest and the infusion intervals were averaged for cardiovascular variables and an average from the last 15 s from the 5th and 10th minute values for TEA.

## 3 RESULTS

Twenty‐seven healthy adults (13 females) participated in the study (mean ± SD: age 59 ± 8 years, height 175 ± 6 cm, weight 83 ± 11 kg, hence body mass index 27 ± 3 kg/m^2^). Cardiovascular variables and blood sample analysis are presented for 26 participants because one participant experienced white fingers during infusion and an increase rather than a decrease in SVR and was therefore excluded for further analysis. Data from 20 participants are presented for impedance data owing to lack of baseline measurement (*n* = 5) and a measurement error (*n* = 2).

The time of blood collection was 117 ± 68 s after the end of infusion (*P* = 0.47 between the two sample times), and there was 313 ± 115 s between the two intervals. The volunteers received 9 ± 2 ml of adrenaline solution for the four periods combined with a peak infusion rate of 30 ± 6 ml/h, corresponding to 0.3 ± 0.2 μg/kg/min.

**FIGURE 2:**
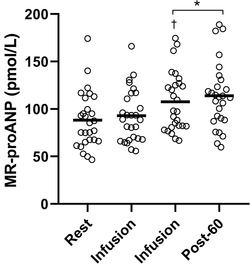
Changes in mid‐regional pro‐atrial natriuretic peptide (MR‐proANP) at rest, during infusions of adrenaline and 60 min after the end of infusion (Post‐60). Open circles indicate individual data, and the horizontal lines indicate the mean values. ^*^ *P* < 0.05 compared with baseline. ^†^ *P* < 0.05 compared with previous time point

**FIGURE 3:**
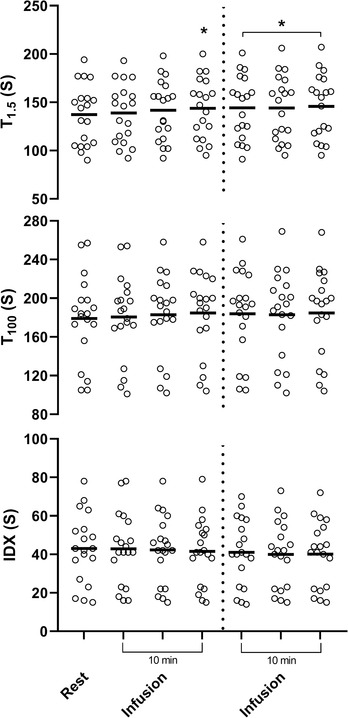
Change in thoracic electrical admittance at 1.5 (*T*_1.5_) and 100 kHz (*T*_100_) at rest and during the four infusions of adrenaline depicted in 5 min segments, with the dotted vertical line indicating the separation between intervals for blood sample collection. IDX is the difference between *T*_1.5_ and *T*_100_ to indicate changes in regional intracellular water. Open circles indicate individual data, and the horizontal lines indicate the mean values. ^*^ *P* < 0.05 compared with baseline

**FIGURE 4:**
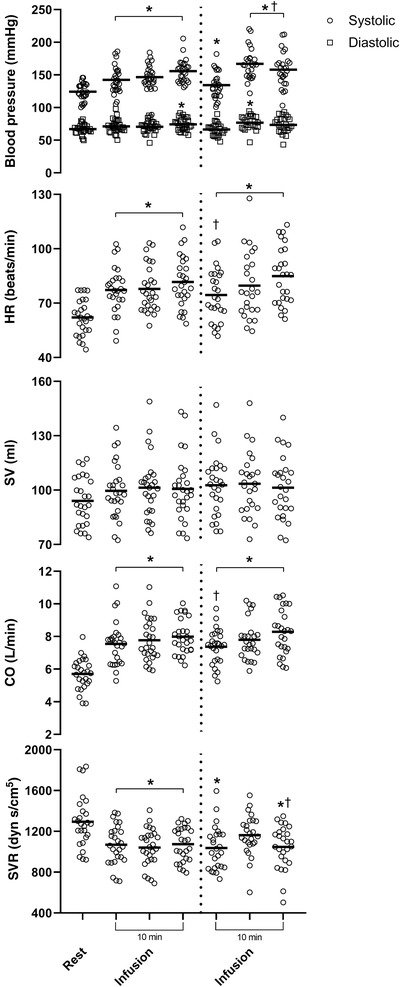
Change in blood pressure (systolic and diastolic), heart rate (HR), stroke volume (SV), cardiac output (CO) and systemic vascular resistance (SVR) at rest and during the four infusions of adrenaline depicted in 5 min segments, with the vertical dotted line indicating the separation between intervals for blood sample collection. Open circles indicate individual data and the horizontal lines indicate the mean values. ^*^ *P* < 0.05 compared with baseline. ^†^ *P* < 0.05 compared with previous time point

There was a marked increase in serum MR‐proANP concentrations (from 88 ± 29 to 108 ± 30 pmol/L) during adrenaline infusion, with the increase being statistically significant during interval 2 (by 26 ± 25%, *P* < 0.001; Figure 2). The increase in serum MR‐proANP concentrations was sustained 60 min after the end of infusion (at 114 ± 34 pmol/L). The increase in MR‐proANP did not result from an increase in CBV, because IDX (−3 ± 17%, *P* = 0.230) and total (regional) body water (*T*_100_: 3 ± 7%, *P* = 0.260) remained similar and yet extracellular (*T*_1.5_) increased (by 5 ± 5%, *P* < 0.005; Figure 3). There was no significant difference for any of the haemodynamic variables between the two periods of adrenaline infusion, and the average is presented (Table 1). Adrenaline infusion increased HR by 33 ± 18% (*P* < 0.005), SV by 6 ± 13% (*P* = 0.028), hence CO (by 42 ± 23%, *P* < 0.005). The HR increased from the start of the infusion, hence CO also increased from the beginning (Figure 4). The MAP increased (by 18 ± 13%, *P* < 0.005), hence SVR decreased (by 14 ± 17%, *P* = 0.006).

**TABLE 1:** Haemodynamic and thoracic electrical admittance variables at rest and during infusion of adrenaline

**Variable**	**Rest**	**Adrenaline**
MAP (mmHg)	88 ± 10	103 ± 14*
HR (beats/min)	63 ± 10	83 ± 14*
SV (ml)	94 ± 13	100 ± 17*
CO (L/min)	6 ± 1	8 ± 1*
SVR (dyn s/cm^5^)	1,268 ± 269	1,067 ± 239*
*T*_1.5_ (S)	138 ± 31	145 ± 32*
*T*_100_ (S)	183 ± 43	187 ± 42
IDX (S)	45 ± 19	42 ± 17

Abbreviations: CO, cardiac output; HR, heart rate; IDX, index value; MAP, mean arterial pressure; SV, stroke volume; SVR, systemic vascular resistance; *T*_1.5_ thoracic electrical admittance at 1.5 kHz; *T*_100_, thoracic electrical admittance at 100 kHz. *Note*. Values are the average of the last minute of rest and of the last minute during the second and fourth infusion intervals. Data are presented as the mean ± SD.

* *P* < 0.05 compared with baseline.

Adrenaline infusion increased venous haemoglobin and plasma sodium, while plasma potassium and calcium decreased. All these variables normalized within 1 h postinfusion, with no change in plasma pH (Table 2). Adrenaline infusion also increased plasma glucose and lactate, with further increases during the second infusion period (both *P* < 0.005), and both remained elevated for 1 h postintervention, while plasma bicarbonate decreased during the two intervals.

**TABLE 2:** Metabolic responses to adrenaline infusion at rest, during four periods of infusion (collected after interval 2 and interval 4) and 1 h postinfusion

**Variable**	**Rest**	**Interval 2**	**Interval 4**	**Post‐60**
MR‐proANP (pmol/L)	88.2 ± 29.1	92.8 ± 27.2	107.6 ± 30.4*, ^†^	113.8 ± 34.3*
pH	7.38 ± 0.02	7.38 ± 0.02	7.38 ± 0.08	7.36 ± 0.03
$P_{CO_{2}}$ (kPa)	6.2 ± 0.7	6.0 ± 0.4	5.9 ± 0.5	6.5 ± 0.6^§^
$P_{O_{2}}$ (kPa)	5.0 ± 2.0	4.9 ± 1.1	4.9 ± 1.1	3.4 ± 1.2*, ^§^
$S_{O_{2}}$ (%)	0.63 ± 0.20	0.66 ± 0.14	0.66 ± 0.16	0.41 ± 0.19*, ^§^
$F_{O_{2}\mathrm{Hb}}$ (%)	0.61 ± 0.20	0.65 ± 0.13	0.65 ± 0.15	0.41 ± 0.18*, ^§^
Hb (mmol/L)	8.7 ± 0.6	8.9 ± 0.6*	8.9 ± 0.7*	8.7 ± 0.8^§^
K^+^ (mmol/L)	4.1 ± 0.3	3.4 ± 0.4*	3.1 ± 0.3*	4.1 ± 0.4^§^
Na^+^ (mmol/L)	141.3 ± 1.6	142.2 ± 1.7*	142.5 ± 1.7*	140.8 ± 2.7^§^
Ca^2+^ (mmol/L)	1.20 ± 0.03	1.21 ± 0.03	1.19 ± 0.03*, ^†^	1.20 ± 0.03^‡^
Glucose (mmol/L)	5.8 ± 1.0	7.1 ± 0.9*	9.0 ± 1.0*, ^†^	7.2 ± 1.3*, ^‡^
Lactate (mmol/L)	1.1 ± 0.3	2.0 ± 0.5*	2.9 ± 0.7*, ^†^	1.6 ± 0.5*, ^§^
HCO_3_^–^ (mmol/L)	24.9 ± 0.9	24.3 ± 1.1*	23.2 ± 1.1*, ^†^	24.4 ± 1.1^†^

Abbreviations: $F_{O_{2}\mathrm{Hb}}$, fraction of oxygenated haemoglobin, Hb, haemoglobin; MR‐proANP, mid‐regional pro‐atrial natriuretic peptide; $P_{CO_{2}}$, carbon dioxide partial pressure; $P_{O_{2}}$, oxygen partial pressure; Post‐60, 60 min after the end of infusion; $S_{O_{2}}$, fraction oxygen saturation. *Note*. Data are presented as the mean ± SD.

* *P* < 0.05 compared with baseline.

^†^ *P* < 0.05 compared with previous interval.

^‡^ *P* < 0.05 compared with interval 4.

^§^ *P* < 0.05 compared with intervals 2 and 4.

## 4 DISCUSSION

This study addressed whether the chronotropic and inotropic effects of adrenaline (Stratton et al., 1985) are reflected in (serum) MR‐proANP concentrations, as reported for plasma ANP (Morrow et al., 1989; Sanfield et al., 1987; Tunny et al., 1988), and reflected in CBV as evaluated by TEA. Adrenaline increased both HR and SV, hence CO, but was accompanied by only a small increase in low‐frequency TEA, whereas serum MR‐proANP increased to the highest level after the end of the infusion.

As expected, SV increased with administration of adrenaline. With the increase in HR, the CO also increased, in accordance with classical investigations (e.g., Barcroft and Konzett, 1949; Freyschuss et al., 1986). Also, studies implementing pulse‐contour analysis during adrenaline infusion (Niemann et al., 2019; Rokamp et al., 2017; Seifert et al., 2009) demonstrate a ∼40–76% increase in CO compared with a 44% increase in the present study using a stepwise rather than a continuous infusion rate and a peripheral rather than a central venous line. The time and dose differ among the studies (0.08–0.01 μg/kg/min), and CO increases ∼2‐fold when the present peak dose of ∼0.3 μg/kg/min is administered for 5 min (Elliott et al., 2014) as adrenaline increases CO in a stepwise fashion to its concentration, for example, from 0.001 to 0.08 μg/kg/min (Freyschuss et al., 1986; Leenen et al., 1988; Maggs et al., 1994). Yet, the present subjects were older than those typically exposed experimentally to adrenaline, and with age, the SV rather than the HR response is blunted (White & Leenen, 1997).

Adrenaline infusion also increases HR in a dose‐dependent manner, emptying the left ventricle, and increases systolic pressure with little change in the diastolic heart volume (Kjellberg et al., 1952; Stratton et al., 1985). However, the left ventricular end‐diastolic dimension has also been observed to increase during adrenaline infusion (Leenen et al., 1988). The CBV, as evaluated by TEA, did not increase on infusion of adrenaline; however, there was an increase in the extracellular thoracic volume as reflected by low‐frequency current TEA that might support CO (González‐Alonso et al., 2006) and is reflected in venous haemoconcentration, and also arterial haemotocrit tends to increase (Rokamp et al., 2017). We found that serum MR‐proANP increased with adrenaline infusion and remained elevated 60 min thereafter. Thus, ANP is not only released in response to changes in CBV, such as during head‐up tilt (Matzen et al., 1990) or as a consequence of cardiac disease (Elmas et al., 2008; Lindberg et al., 2015; Moertl et al., 2009), but also by infusion of adrenaline (Morrow et al., 1989; Sanfield et al., 1987; Tunny et al., 1988), apparently independent of CBV.

Adrenaline has widespread metabolic actions, and infusion of adrenaline decreased plasma potassium and calcium in accordance with the work of Hansen et al. (1990). Also, catecholamines (e.g., during whole‐body exercise) stimulate liver gluconeogenesis and muscle glycogenolysis (Kjær et al., 1987); therefore, the increase in plasma glucose and lactate in response to adrenaline is to be expected.

ProANP has 126 amino acids and a longer half‐life than ANP and is therefore suggested to be a more reliable analyte (Buckley et al., 1999). However, proANP_1–98_ is exposed to further fragmentation (Cappellin et al., 2001; Morgenthaler et al., 2004), and immunoassays have been developed to target different regions. The right atrium becomes smaller when venous return to the heart is limited, for example, by manipulating CBV, and plasma ANP decreases during lower‐body negative pressure (Cai et al., 2000b), such as during head‐up tilt (Matzen et al., 1990) and during sitting and standing (Vogelsang et al., 2006). Yet, ANP fragments respond in different ways to CBV. Athletes demonstrate an enlarged CBV (Sawka et al., 2000), and their proANP_(31–67)_ is elevated, although proANP_(1–30)_ is not (De Palo et al., 2000). Conversely, proANP_(1–30)_ rather than proANP_(31–67)_ responds to acute exercise, reflecting that proANP_(1–30)_ has a shorter degradation time than proANP_(31–67)_ (Nielsen et al., 2001). When targeting the mid‐region of proANP (amino acids 53–90), the analysis is correlated with perioperative fluid balance and follows a compromised CBV during laparoscopic procedures including head‐up tilt (Strandby et al., 2017), open cystectomy (Rasmussen et al., 2016) and open oesophagectomy (Strandby et al., 2019a). Conversely, MR‐proANP does not follow changes in haemorrhage‐induced hypovolaemia in pigs, with or without thoracic epidural anaesthesia (Strandby et al., 2019b). Haemorrhagic shock increases plasma adrenaline (e.g., Jacobsen et al., 1990), possibly explaining an increase in MR‐proANP. Also, when conducting maximal (rather than submaximal) exercise, the large increase in adrenaline could contribute to the increase in plasma ANP (Perrault et al., 1989), because adrenaline infusion results in similar levels to those during physiological stress with similar changes in SV, ejection fraction and SVR (Stratton et al., 1985). Concomitant with positive inotropy and chronotropy, exercise also increases CBV by the muscle pump, thereby increasing ANP (Vogelsang et al., 2006; Yoshiga et al., 2019), and physiological stress imposed by passive heating results in a correlation between changes in CBV and ANP (Vogelsang et al., 2012).

In the present study, serum MR‐proANP remained elevated 1 h after adrenaline infusion. Tunny et al. (1988) found that venous plasma ANP reached resting values 30 min after the infusion of adrenaline. Likewise, Perrault et al. (1989) found that both venous plasma ANP and adrenaline levels returned to resting values 30 min after termination of maximal ergometer cycling. However, plasma proANP_1–30_ remained elevated 30 min after the end of maximal exercise (Engelmann et al., 2005). When manipulating CBV by head‐up tilt, arterial plasma ANP did not reach resting values 30 min after a return to supine rest (Matzen et al., 1990), whereas arterial plasma ANP returned to the resting level 10 min after lower‐body negative pressure was terminated (Cai et al., 2000b). Thus, plasma ANP has a short half‐life, whereas the half‐life of different prohormones is prolonged to various extents, as also illustrated by Baker et al. (1991), who found that ANP returns to the resting level within 30 min, whereas proANP remains elevated 1 h after maximal cycling exercise.

A limitation to the present study is the peripheral site of infusion, which, potentially, affected the even distribution of adrenaline. Also, other variants of ANP could have been determined and, for example, echocardiography could have been used to evaluate filling of the atrium during the infusion.

In conclusion, given that CBV remained similar, serum mid‐regional proANP is not only influenced by CBV, but also responds to the chronotropic and inotropic state of the heart induced by adrenaline infusion, or adrenaline might directly induce release of ANP variants from the myocytes. Yet, for example, central hypovolaemia (e.g., head‐up tilt or lower‐body negative pressure) is associated with elevated catecholamines at the same time as ANP fragments decrease in plasma.

## COMPETING INTERESTS

None declared.

## AUTHOR CONTRIBUTIONS

Casper Sejersen, Jonathan J. Bjerre‐Bastos, Henning B. Nielsen, Asger R. Bihlet and Niels H. Secher designed the study. Casper Sejersen and Jonathan J. Bjerre‐Bastos conducted the experiment and collected the data. Casper Sejersen, Jens P. Goetze and Niels H. Secher analysed and interpreted the data. Casper Sejersen and Niels H. Secher drafted the work. All authors critically revised the manuscript, approved the final version and agree to be accountable for all aspects of the work in ensuring that questions related to the accuracy or integrity of any part of the work are appropriately investigated and resolved. All persons designated as authors qualify for authorship, and all those who qualify for authorship are listed.

## Supporting information

Statistical Summary Document

## Data Availability

The data that support the findings of this study are available from the corresponding author upon reasonable request.
